# Cases of the Termination of Acute Rheumatism in Suppuration

**Published:** 1851-03

**Authors:** 


*Cases of the Termination of Acute Rheumatism in Suppuration.* By MM. Fleury, Andral, and Trousseau.—In a late discussion at the French Académie de Médicine on the subject of acute rheumatism, some of the speakers laid considerable stress upon the rarity of the termination of rheumatism in suppuration, and this has led to the publication in the medical journals of some examples of it. Professor Fleury, of Clermont, states that, up to 1848, he had always believed with M. Chomel, that rheumatism never so terminated; for, practising in a part of the country wherein the disease is of frequent occurrence, he had never met with a case of such before, although he had frequently had cases transferred to his surgical wards from the physicians' under the idea that such was the case. The present case occurred in a youth, æt. 18, of sound constitution, and hitherto of good health. He was admitted into the Hôtel-Dieu, at Clermont, 2d October, on account of acute rheumatism of the shoulder and knee, induced by sleeping in a damp chamber. Suppuration was set up, notwithstanding active antiphlogistic treatment, in both joints, the abscesses being left to discharge themselves; and he died on the 12th of November, of purulent infection. On examination, the articular surfaces were found denuded, a metastatic abscess existed in the right lung, but no other disease of any of the viscera was found.

M. Andral read the next case to the Academy. A woman, æt. 67, of feeble constitution, was admitted into La Charité early in July last, suffering from pneumonia, from which she rapidly recovered by bleeding and tartar emetic, and was in a state of convalescence, when, from the effect of a current of air, she became attacked with acute rheumatism of the shoulders, unattended with complication, the attendant fever being great. She was bled, which so enfeebled her as to forbid the repetition; and next day twelve grains of quinine in the twenty-four hours were prescribed. The disease proceeded on to a fatal termination with fearful rapidity, without any complication occurring, any anormal sound of the heart, or any other joint participating. She died on the eighth or ninth day, having exhibited no other symptom than intense pain in both shoulders, a pulse of constantly increasing frequency, and a state of general anguish and rapid sinking, resembling that observed in acute peritonitis. The most careful examination of every organ failed to elicit any explanation of the issue; nor were there any signs of phlebitis or purulent infection. Both shoulder-joints, however, were found filled with white, homogeneous pus, and the synovial membrane was of the intensest red, the articular cartilage retaining its normal colour. Some of the bursæ in communication with the joints were also filled with pus; but external to the joints all was normal; bones, periosteum, and muscles having undergone no change whatever.

M. Trousseau relates a case also, which occurred in a child æt. 9, who, having always enjoyed good health, and been unexposed to privations, was attacked with intense scarlatina, and, during its prevalence, various of the joints became the seat of acute rheumatism. She died on the fifth day of the scarlatina, and third of the rheumatism. On examination, the various organs were found in a healthy condition, with the exception of the pleura, which contained a considerable quantity of serosity. Both shoulder-joints, and those of the elbows, knees, and ankles, were filled with a considerable quantity of greenish, well-formed pus, accompanied by considerable vascularity of the subsynovial cellular tissue.

M. Trousseau observes, that the practitioners who have much observed disease in children, must be aware what a powerful effect the exanthemata exert in engendering a *purulent diathesis.* If this child had contracted the rheumatism unconnectedly with the scarlatina, it would have probably determined the synovial effusion usually found; but the scarlatina having changed the *crasis* of the blood, developed the suppurative diathesis, and converted a trifling affection into one of an irremediable character. Was not the old woman, whose case is related by M. Andral, reduced, M. Trousseau inquires, by the debilitating treatment to which she was subjected, to a condition analogous to that into which scarlatina so often places children?—*British and Foreign Review, January* 1851, *from Bulletin de l'Académie,* xv. pp. 774–785; *Gazette Médicale,* Nos. 26 and 32; *L'Union Médicale,* No. 102.

